# Machine Learning-Based Fault Detection and Exclusion for Global Navigation Satellite System Pseudorange in the Measurement Domain

**DOI:** 10.3390/s25030817

**Published:** 2025-01-29

**Authors:** Ma’mon Saeed Alghananim, Cheng Feng, Yuxiang Feng, Washington Yotto Ochieng

**Affiliations:** Department of Civil and Environmental Engineering, Imperial College London, Skempton Building, South Kensington, London SW7 2BU, UK; cheng.feng18@imperial.ac.uk (C.F.); y.feng19@imperial.ac.uk (Y.F.); w.ochieng@imperial.ac.uk (W.Y.O.)

**Keywords:** GNSS, fault detection and exclusion, machine learning

## Abstract

Global Navigation Satellite Systems (GNSS) support numerous applications, including mission-critical ones that require a high level of integrity for safe operations, such as air, maritime, and land-based navigation. Fault Detection and Exclusion (FDE) is crucial for mission-critical applications, as faulty measurements significantly impact system integrity. FDE can be applied within the positioning algorithm in the measurement’s domain and the integrity monitoring domain. Previous research has utilized a limited number of Machine Learning (ML) models and Quality Indicators (QIs) for the FDE process in the measurement domain. It has not evaluated the pseudorange measurement fault thresholds that need to be detected. In addition, ML models were mainly evaluated based on accuracy, which alone does not provide a comprehensive evaluation. This paper introduces a comprehensive framework for traditional ML-based FDE prediction models in the measurement domain for pseudorange in complex environments. For the first time, this study evaluates the fault detection thresholds across 40 values, ranging from 1 to 40 m, using six ML models for FDE. These models include Decision Tree, K-Nearest Neighbors (KNN), Discriminant, Logistic, Neural Network, and Trees (Boosted, Bagged, and Rusboosted). The models are comprehensively assessed based on four key aspects: accuracy, probability of misdetection, probability of fault detection, and the percentage of excluded data. The results show that ML models can provide a high level of performance in the FDE process, exceeding 95% accuracy when the fault threshold is equal to or greater than 4 m, with KNN providing the highest FDE performance.

## 1. Introduction

Global Navigation Satellite Systems (GNSS) can provide position, navigation, and timing (PNT) information for users worldwide and support a wide range of applications. For instance, London Economics [1] estimated that the economic benefit of GNSS to the UK is GBP 6.7 billion per annum, and the economic impact of a five-day disruption of GNSS in the UK could be as high as GBP 5.2 billion. In addition, GNSS has the potential to offer many more benefits in the future, as PNT technology has become significant in numerous applications today.

Some GNSS applications require a high level of safety/integrity including aviation, maritime, autonomous driving, rail, and farming. The positioning algorithms in these applications should account for extreme events, including faulty measurements and their impact on the positioning solution. Therefore, Fault Detection and Exclusion (FDE) is of particular importance to these applications. In addition to mission-critical applications, some non-mission-critical applications of GNSS also require a high level of positioning integrity, such as precise surveying. The ‘Study of Critical Dependencies’ by the Government Office for Science [2] recommended an integrity risk of $10^{-6}$ for surveying, with centimeter to decimeter accuracy levels. Therefore, the development of industrial-grade precise GNSS devices aims to achieve a high level of reliability (≥0.99%) (e.g., Leica Geosystems [3]).

The FDE process is applied within two main phases in the positioning algorithm at the user level: the measurement domain and the Receiver Autonomous Integrity Monitoring (RAIM). Starting with the latter, RAIM is a technology developed to assess the integrity of GNSS signals within a GNSS receiver system. It has two main functions: FDE and raising an alarm when a system should not be used. Conventionally, the FDE stage within RAIM represents the central FDE process within the positioning algorithm.

The main output of RAIM is the protection level, which refers to the upper bound that a position error must not exceed without being detected with a given probability (integrity risk). The protection level is a function of, but not limited to, FDE, position uncertainty, overbounding methodology, and the pre-processing chain. FDE is important in protection level computation, as it is linked directly with PL equations and indirectly through error distribution overbounding, which is significantly affected by faulty measurements [4]. Consequently, FDE plays a critical role in positioning algorithms, effectively minimizing both the protection level and the positioning uncertainty.

In RAIM algorithms, the FDE process is based on the test statistic(s). Classical RAIM [5,6,7,8] is based on a residuals-based test statistic, which is limited in its ability to detect multiple simultaneous failures. Therefore, the Solution Separation (SS) method [5,7,8] is developed to detect multiple simultaneous failures. The idea of the SS method is to create satellite subsets by excluding one or more satellites, considering the probability of missed detection. Then, test statistics are implemented for the created satellite subsets to detect failures. Advanced RAIM (ARAIM) [9,10,11] is developed as an SS-based RAIM algorithm due to its potential to detect multiple simultaneous failures using multi-GNSS positioning.

However, ARAIM is very complex in implementation; therefore, several efforts have been made to simplify ARAIM by creating subset solutions. One approach is based on selecting subset solutions based on Geometric Dilution of Precision (GDOP) values [12,13,14,15]. Another approach focuses on using one or more quality indicators (e.g., elevation angles) in combination with Dilution of Precision (DOP) values [16]. In addition, there are system-level approaches based on Satellite-Based Augmentation System (SBAS) information and/or Integrity Support Message (ISM) parameters [17,18].

However, approaches based on DOP values are generally complex to implement in real-time applications, as they involve creating subset solutions in real-time and subsequently selecting the most suitable subset solution based on the DOP value. In addition, DOP is an indicator of positioning performance, but by itself, it cannot precisely define the subset solutions. While the SBAS/ISM parameters approach has limitations in dealing with user-level outliers.

In addition to the FDE process within RAIM, FDE at the measurement domain can introduce an additional FDE layer within the positioning algorithm. This layer can enhance system integrity, particularly at the user level in complex environments. Moreover, this layer can be utilized to reduce the satellite subsets, leading to simplified ARAIM complexity. The importance of this layer lies in its ability to address outliers at the user level, which is not accounted for within SBAS. Principally, this layer becomes more important with increasing complexity in dynamic mode in complex environments.

The FDE in the measurement domain is based on detecting faulty measurements using Quality Indicators (QIs) such as satellite elevation, signal-to-noise ratio, and multipath indicators. The correlation between the QIs and GNSS measurement quality is nonlinear and high-dimensional. This suggests using Machine Learning (ML) to link the QIs with measurement quality. Therefore, several studies have utilized ML within the GNSS positioning algorithm. Ref. [19] developed an ML-based signal spoofing detection using Support Vector Machines (SVMs). Ref. [20] employed an integration of Fully Connected Neural Networks (FCNNs) and Long Short-Term Memory (LSTM) networks to make predictions on GNSS satellite visibility and pseudorange error in urban areas.

However, there is a notable lack of studies exploring ML-based FDE in the measurement domain. Previous studies did not directly focus on FDE and only utilized a limited number of ML methods to create a prediction model. Moreover, no studies have evaluated all aspects of FDE comprehensively, as accuracy is insufficient to evaluate the model’s performance. Additional aspects must also be considered, including the probability of missed detection, the probability of false detection, and the percentage of detected outliers.

In mission-critical applications, the probability of missed detection is significantly important as it relates to risk and should be accounted for in the computation of the protection level. The probability of false detection represents the level of loss of clean data from the ML models, and the percentage of excluded data includes the total loss of measurements, both clean and non-clean. This is critical for understanding the ratio of measurements used in the positioning algorithm. In addition, no study has linked the outlier thresholds to model performance. This threshold refers to the outlier values that need to be detected, which are a function of application requirements. As selecting the best threshold significantly impacts model accuracy, as well as the probabilities of false detection and missed detection, sensitivity analysis is required for the ML-based FDE models to define the ideal threshold. Put differently, in the FDE process, the problem expands beyond defining the best ML model and QIs to include determining the optimal outlier threshold.

Advanced ML techniques, such as SVM and sophisticated deep learning models—like spatio-temporal graph neural networks and transformer-based architectures—offer the potential for enhanced accuracy by capturing intricate, non-linear patterns. Even though these approaches can outperform traditional ML models, they pose significant challenges for real-time implementation. Convergence often proves difficult when high data complexity combines with large-scale datasets, making these methods less practical for real-world GNSS applications. Furthermore, the aforementioned limitations of current ML models remain unresolved in traditional ML. Therefore, resolving these issues in simpler models might serve as the first step toward adopting more complex approaches.

This paper conducts a comprehensive evaluation for the first time of traditional ML models across 40 thresholds (between 1 and 40 m) for FDE of pseudorange measurements in dynamic mode complex environments. This includes utilizing six ML models: Decision Tree, K-Nearest Neighbors (KNN), Discriminant, Logistic, Neural Network, and trees (Boosted, Bagged, and Rusboosted). These models offer advantages such as computational efficiency, scalability, and suitability for the practical constraints of large datasets. To define the model configuration parameters, 640 ML models have been created across the 40 thresholds using various configuration parameters.

The six ML models across the 40 threshold values are assessed in four key aspects: accuracy, probability of missed detection, probability of false detection, and the percentage of detected outliers. The ML models in this paper are based on 11 QIs, including carrier-to-noise ratio, multipath standard deviation indicators, phase lock, phase lock time, satellite elevation, GNSS constellation ID, signal ID, speed, code lock, code lock time, half cycle, and satellite elevation.

## 2. Methodology and Evaluation Framework

### 2.1. Methodology Overview

**Figure 1:**
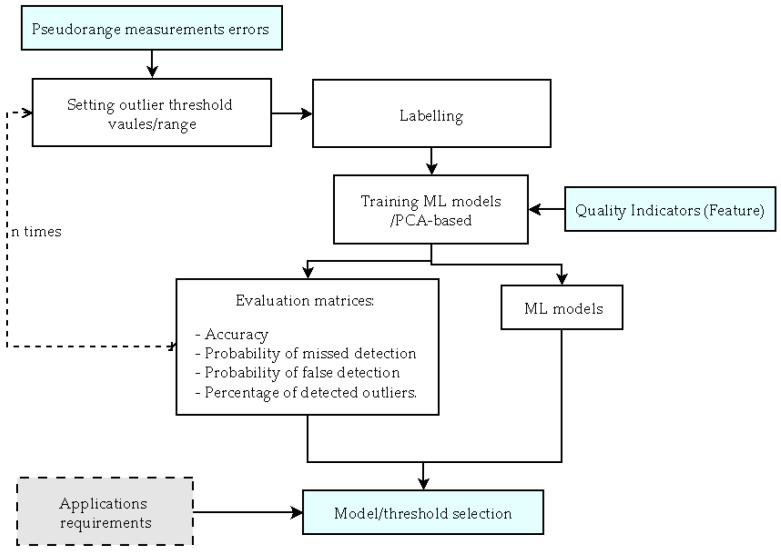
The architecture of the ML-based FDE methodology for GNSS measurements.

Figure 1 presents the ML-based FDE methodology used in this paper. The methodology framework aims to identify the best-performing ML models across various failure thresholds. The initial step involves identifying failure threshold values (range) based on the characteristics of the raw data. Within each threshold, pseudorange measurement errors are labelled as outliers or non-outliers. Using the labelled residuals and QIs, ML models are trained for the FDE process.

From all the ML model results across different thresholds, the best models can be selected, taking into account the application’s requirements. Section 2.2 summarizes the QIs utilized in this paper, while Section 2.3 provides an overview of the ML models employed in this study. Section 2.4 presents a detailed description of the validation metrics used in this paper.

### 2.2. Quality Indicators

The following QIs are used in this paper, along with their descriptions:

- The GNSS constellation ID (e.g., GPS, GLONASS) represents the specific GNSS con-stellation to which the satellites belong.
- Signal ID (e.g., GPS L1 and GLONASS L1OF).
- The carrier-to-noise ratio (C/N0), expressed in dB-Hz, measures the strength of the GNSS signal. A higher C/N0 indicates a stronger signal, which can provide more ac-curate positioning than weaker signals. Techniques such as Narrow-band Wide-band Power Ratio (NWPR)-based C/N0 estimation, Signal-to-Noise Power Ratio (SNPR)-based methods, and Signal-to-Noise Variance Ratio (SNVR)-based meth-ods are used to compute C/N0 [21].
- Code lock refers to the state where a GNSS receiver has successfully synchronized with the Pseudorandom Noise (PRN) code transmitted by a satellite. This is closely linked with signal strength or high levels of interference. The code lock is obtained through correlation, a signal processing operation where the receiver matches the in-coming satellite signal with a locally generated replica of the PRN code.
- Code Lock Time, expressed in seconds, refers to the duration it takes for the receiver to achieve synchronization with the PRN code of a GNSS satellite signal. It is measured by tracking the elapsed time from when the receiver starts receiving the signal to when it achieves code lock.
- Multipath Standard Deviation indicates the level of distortion in the signals caused by multipath effects. A zero value suggests no detectable multipath distortion, while higher values indicate increasing levels of multipath. The Multipath Estimating Delay Lock Loop (MEDLL) is a classical multipath mitigation technique for GNSS receivers [22,23,24]. The standard deviation of the estimated multipath signal over time is computed as the multipath standard deviation.
- Phase lock refers to the state where a GNSS receiver has synchronized with the carrier phase of a satellite’s signal. It can be described by categorized values that indicate whether the receiver has achieved phase lock or not. When achieved, this indicates higher signal quality, especially for carrier phase positioning.
- Phase Lock Time refers to the time duration from when the GNSS receiver starts tracking a satellite’s signal to when it achieves a stable lock on the carrier phase of that signal. This involves synchronizing the phase of the receiver’s internal oscillator with the phase of the received satellite signal.
- The “Half Cycle Valid” quality indicator shows the status of half-cycle ambiguities in carrier phase measurements. The receiver monitors the carrier phase measurements for abrupt changes or discontinuities, often referred to as cycle slips or half-cycle slips.
- Satellite elevation presents the satellite’s elevation angle. Satellites with a higher elevation angle can provide better signal quality due to less atmospheric impact and multipath. However, satellites with a lower elevation also have advantages in improving the geometry. Thus, this factor should be considered to strike a balance between geometry and signal quality. Satellite elevation is calculated as the angle between the vector from the receiver to the satellite and the vector perpendicular to the reference system ellipsoid.
- Speed refers to the object’s speed or vehicle speed in cases where GNSS is used in a vehicle, or more generally, the GNSS device’s speed. The accuracy of the po-sitioning decreases with increasing speed due to the difficulties in tracking the GNSS satellites. Speed can be estimated using Doppler shift, position differencing methods, or a combination of both.

### 2.3. Machine Learning Models

This section provides a summary of the ML models utilized in this paper, detailed in Section 2.3.1, Section 2.3.2, Section 2.3.3, Section 2.3.4, Section 2.3.5 and Section 2.3.6. While all these models are tested in this paper, Section 2.3.7 offers an initial investigation into the candidate models that could potentially deliver the highest performance, based on the characteristics of the models and data.

#### 2.3.1. Decision Tree

Decision tree in classification is based on creating a tree-like structure that predicts the class label of a given data instance based on its features/QIs. The process of building a decision tree involves recursively partitioning the dataset into subsets based on feature values. At each internal node of the tree, a feature and a threshold are selected to split the data. The splitting criterion aims to maximize the homogeneity or purity of the resulting subsets with respect to the class labels. An important hyperparameter is the maximum number of splits, which directly influences the depth of the decision tree. Setting this hyperparameter appropriately is essential to achieving optimal model performance. If this value is set too high, it may result in overfitting, where the model learns to capture noise and idiosyncrasies in the training data, leading to poor generalization on unseen data. Conversely, a small value can improve robustness and interpretability but may suffer from lower training accuracy [25].

To address these concerns and explore the trade-offs associated with different hyperparameter values, various types of decision trees can be employed to investigate the impact of tree depth on prediction performance. This empirical analysis aims to identify the most suitable configuration that strikes a balance between model complexity, interpretability, and accuracy.

#### 2.3.2. K-Nearest Neighbors

KNN operates on the premise that similar data points are often in close proximity. The KNN classification approach assigns a label to the new data point based on the majority label of the neighbors. There are three main critical configuration parameters for KNN, which include the number of neighbors (k), the choice of distance metric (e.g., Euclidean or Manhattan distance), and the decision between a weighted or unweighted approach. The prediction relies on k neighbors and is based on a distance metric (e.g., Euclidean or Manhattan distance).

This approach often yields satisfactory performance, showcasing notable advantages in terms of simplicity, interpretability, and resilience against noisy data [26]. However, it is important to note that this method is susceptible to variations in the parameter k and entails a high computational burden [27].

#### 2.3.3. Discriminant

As a classic supervised technique for data classification, the main objective of Discriminant Analysis is to model the distribution of features within each class and use this information to compute discriminant functions. These functions act as decision boundaries and are used to classify new data instances into their respective classes. Discriminant classifiers are easy to compute, inherently support multiclass classification, and do not require tuning of hyperparameters [28].

There are two main types of Discriminant Analysis: Linear Discriminant Analysis (LDA) and Quadratic Discriminant Analysis (QDA). The former is based on finding a linear combination of features that best separates the classes and assumes that the data are normally distributed with the same covariance matrix for each class. In contrast, the latter identifies the quadratic decision boundary that best separates the classes and does not assume equal covariance matrices for each class. However, it also assumes that the data are normally distributed.

#### 2.3.4. Logistic Regression

As a popular and fundamental statistical method used for binary classification tasks, logistic regression models the relationship between a set of input features and a binary output variable, where the output variable represents one of two classes. The logistic function maps the linear combination to a value between 0 and 1, representing the probability of the data instance belonging to the positive class (class 1) [29]. The probability of the data instance belonging to the negative class (class 0) is then obtained by subtracting this probability from 1.

Logistic regression can be classified as traditional logistic and kernel logistic. The kernel logistic uses the kernel trick to transform the input space into a higher-dimensional space where linear separation is possible, thereby handling the non-linearity in the data. However, the kernel logistic is more complex than traditional logistic regression, and the transformation into a high-dimensional space adds complexity to the direct relationship between features and the target variable.

#### 2.3.5. Neural Network

Artificial neural networks are composed of interconnected nodes and organized in layers. The depth of the network is determined by the number of hidden layers. Each neuron in a layer is connected to every neuron in the subsequent layer. These connections are governed by weight parameters, signifying their significance in the network’s learning process. Specifically, the interconnected neurons apply a weight matrix multiplication to the input, followed by the addition of a bias vector, contributing to the network’s ability to capture intricate patterns and relationships within the data. Moreover, the activation function, serving as a critical nonlinearity element, is subsequently applied after each fully connected layer. Activation functions introduce nonlinear transformations to the network’s output, allowing it to approximate complex decision boundaries in the data and facilitating feature learning. Finally, the last fully connected layer, together with the subsequent softmax activation function, culminates in producing the network’s final classification scores [30].

#### 2.3.6. Ensemble

Ensemble classifiers represent a significant paradigm in the field of Machine Learning, wherein multiple individual classifiers, known as base models, are harnessed to collectively enhance prediction accuracy and robustness. The core principle underlying ensemble methods lies in synergistically integrating the diverse expertise of constituent models [31]. The ensemble combines the prediction from multiple models.

There are several types of ensemble classification, including but not limited to Bagging, Boosting, and Random Under Sampling (RUSBoost). Bagging involves training multiple models on different random subsets of the training data, created through bootstrapping—a process of sampling with replacement from the training set. This technique is particularly effective in reducing overfitting and variance in models. On the other hand, Boosting takes a sequential approach to model training, where each new model is trained to focus on and correct the errors made by the preceding models. This method is adept at increasing the predictive accuracy of an ensemble by reducing bias. RUSBoost, a specialized variant of Boosting, is tailored to tackle the challenges posed by imbalanced datasets. By integrating the principles of random under-sampling of the majority class into the boosting framework, RUSBoost effectively balances the training process, ensuring that the minority class is adequately represented and learned by the model. Each of these techniques, with their distinct methodologies, plays a crucial role in the realm of ensemble learning, enhancing the performance of predictive models across various scenarios.

#### 2.3.7. Candidate Machine Learning Models for the FDE Process

In the evaluation of candidate Machine Learning models for a classification task involving 11 QIs as input features, the choice of the most appropriate model becomes pivotal. This discussion aims to assess the suitability of the aforementioned models in the context of this specific task.

Logistic Regression and Discriminant Analysis are traditionally popular for their simplicity and efficiency. However, their fundamental assumption of a linear relationship between independent variables and the dependent variable poses a significant limitation in this context. The complexity and potential non-linear interactions amongst the 11 QIs are likely to be inadequately modelled by these methods. Logistic Regression, in particular, may struggle with the intricate interplay of the features, which is a crucial aspect of this task.

Decision Trees, known for their interpretability and ease of use, offer a more intuitive approach. However, their performance is hampered when dealing with complex relationships between a higher number of features. In our case, the Decision Trees’ inherent simplicity might not suffice to unravel the complexity embedded in the 11-dimensional input space, leading to potentially suboptimal performance.

On the brighter side, KNN emerges as a strong contender. Its non-parametric nature allows it to operate effectively without assumptions about data distribution, a significant advantage considering the diversity of the 11 QIs. KNN’s ability to classify samples based on the similarity to their nearest neighbors is particularly suited for a dataset where patterns of similarity strongly indicate class membership. In addition, concerns about the curse of dimensionality are mitigated in this scenario, possibly due to the dataset’s characteristics that align well with KNN’s strengths.

Bagged Trees offer a robust solution by amalgamating multiple decision trees, each trained on different data subsets. This ensemble approach not only captures complex, non-linear relationships but also reduces the risk of overfitting, a typical concern with single decision trees. The presence of noise or outliers in the dataset, which is not uncommon in real-world scenarios, is effectively managed by this method, enhancing the model’s reliability and robustness.

Neural networks stand out for their ability to model intricate, non-linear interactions through their layered architecture. They excel in learning effective representations of data, a feature crucial for managing the complexities of the 11 QIs. However, this power comes with the need for extensive tuning and a substantial dataset to train effectively. The performance of neural networks in this context would hinge on the adequacy of tuning and the quality of the data, making it a potentially high-performing yet resource-intensive option.

### 2.4. Evaluation Metrics

The evaluation metrics used in this paper include the ML models’ accuracy, probability of missed detection, probability of false detection, and percentage of the excluded data. These metrics have been used to assess the models in four aspects: overall performance, risks associated with the models, percentage of lost clean data, and percentage of excluded data in the measurement domain. The following summarizes these metrics:

- Accuracy: indicates the overall performance of the models, which is a function of the probabilities of missed detection and the probability of false detection. In this paper, cross-validation accuracy is used, as tests show that it is nearly equivalent to testing a 20% subset set aside in the data. Therefore, cross-validation is employed to maximize the dataset used for testing.
- The probability of missed detection refers to the probability that the model predicts measurements as clean, which are not. This error is important for missioncritical applications, as it is associated with the risk aspect that should be accounted for in protection level computation.
- The probability of false detection, also referred to as False Positive Rate, refers to the probability that the model predicts measurements as faulty while they are clean. This is linked with the percentage of lost clean data aspect.
- Percentage of excluded data, which includes the total loss of clean and non-clean measurements. This is crucial for understanding the ratio of measurements that will be used in the positioning algorithm. The percentage of excluded data should be within low limits, as excluding a high percentage impacts the model’s performance by reducing the number of satellites and impacting the geometry. Faulty measurements from satellites with low elevation have a higher probability of failure, and by excluding these satellites, the geometry is impacted. Thus, this stage aims to detect a potential failure at an early stage, and further FDE can then be applied in the position domain. It is important to highlight that the models with the highest accuracy are not always the ones with the lower probability of missed detection or false detection. Thus, these aspects should be evaluated, and the models can then be selected based on these aspects, accounting for the processing power of these models.

## 3. Results

### 3.1. Data Exploratory

The data were collected in dynamic mode and in various environments using a vehicle-mounted multi-constellation GNSS receiver (U-blox’s F9 receiver) and Trimble’s Applanix reference system. Position, velocity, heading, and accuracy data were generated using the Applanix reference system and processed with Applanix post-processing software (POSPac 8), achieving an average horizontal accuracy of 4 cm and vertical accuracy of 6 cm across all datasets.

**Figure 2:**
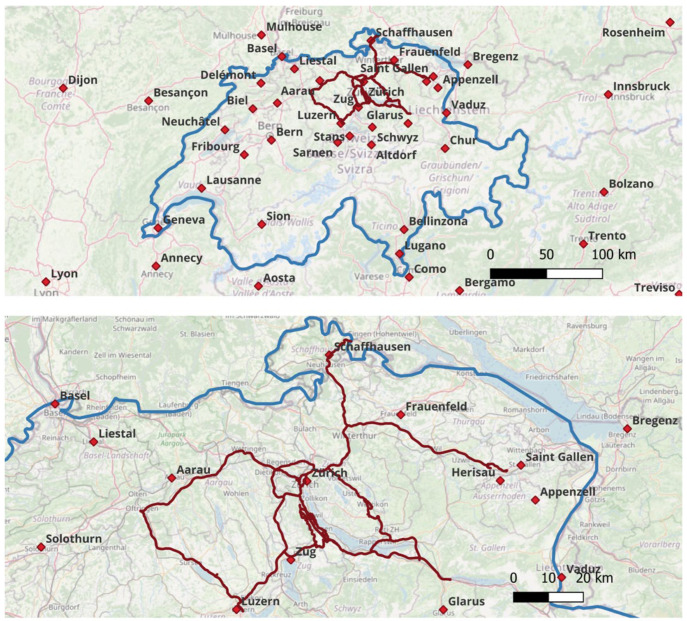
Map of the data collection route (~3455 km) used in this study in Switzerland. The blue line represents the Swiss border, while the red path indicates the vehicle’s route.

Data collection was carried out in Switzerland, spanning a route of approximately 3455 km through various cities and regions, as illustrated in the map presented in Figure 2. The data encompass a variety of environments: highways represent open-sky conditions, sometimes bordering on semi-urban areas, while cities like Zürich and Zug exemplify urban environments. The vehicle maintained an average speed of about 70 km/h, with a minimum of 42 km/h and a maximum of 142 km/h.

**Figure 3:**
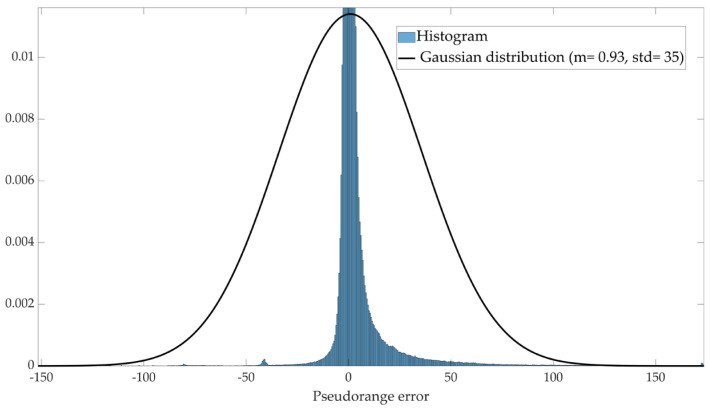
Fitted Gaussian distribution of pseudorange errors across 4,532,170 rows.

Due to the high level of accuracy of the reference system, the measurements it provided were considered as true values. Measurement errors were subsequently estimated by comparing the data from the GNSS receiver with that from the reference system. The dataset combined 26 subsets, totaling 4,532,170 rows, collected on 26 different days and under various operational conditions. The standard deviation of the pseudorange error, calculated across the 4,532,170 rows, is 35 m. This value is significantly affected by outliers, as shown in Figure 3, which presents the Gaussian distribution fitted to the dataset.

The data also include the 11 Quality Indicators (QIs), as described in Section 2.2. Table 1 summarizes the numerical QIs, providing their mean and standard deviation. Table 2 summarizes the categorical QIs, detailing their categories and respective percentages.

Pearson correlation has been used to investigate the correlation between QIs, as shown in Table 3. The Pearson results indicate a high level of correlation between certain QIs, such as C/N0 and three other QIs (phase lock, half cycle valid, and elevation). This suggests utilizing Principal Component Analysis (PCA) with ML, as it can capture the correlation structure among QIs, allowing for the development of more efficient ML models. However, Pearson correlation is based on a linear assumption and can be used mainly to gain a general overview of the correlations between the variables.

**Figure 4:**
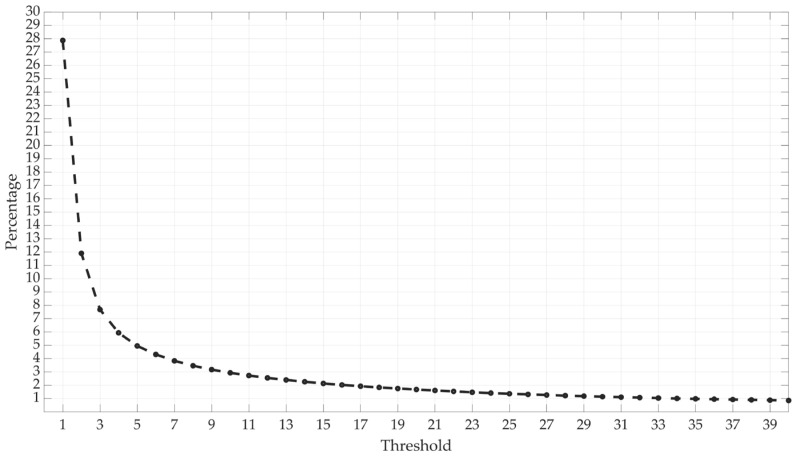
The relationship between the threshold and the proportion of data exceeding that threshold.

The threshold is set by identifying an initial percentage for the target faulty values, which are set within a range of 1 to 40 m. This represents target faulty values for approximately 1% to 28% of the datasets, as shown in Figure 4. The selection can then be adapted on the application side according to the specific requirements of the application. While lowering the thresholds enhances data quality, it also results in a higher loss of data.

### 3.2. Model Selection and Configuration Optimization for Machine Learning Approaches

**Figure 5:**
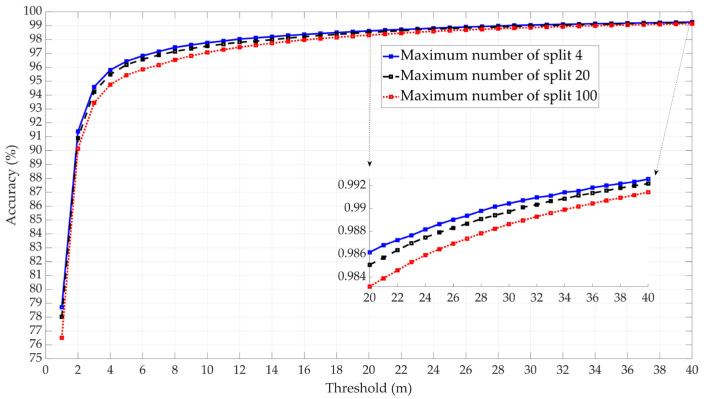
Comparative performance of decision tree model with three maximum split numbers.

The configuration parameters for the six ML models were chosen after evaluating the models’ performance across various settings. Beginning with the Decision Tree, the model was tested using three different maximum numbers of splits: 4, 20, and 100. The highest accuracy was achieved with a maximum of 4 splits, which exhibited marginally better performance than using 20 splits, as shown in Figure 5. Therefore, the decision tree model with a maximum of four splits has been selected as the candidate decision tree model.

**Figure 6:**
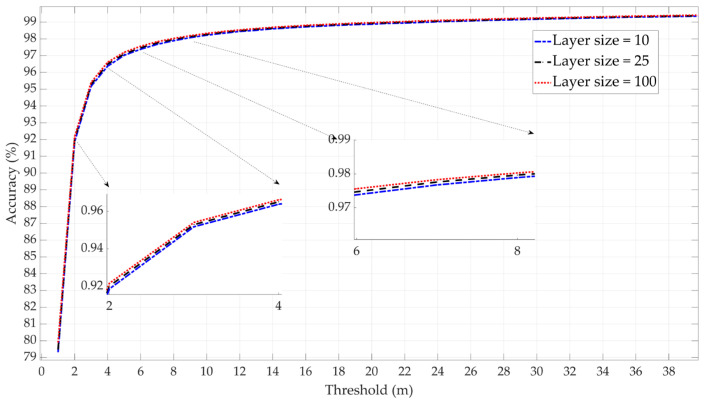
Comparative performance NN model with different layer sizes (10, 25, and 100).

In the NN tests, single-layer networks with three different layer sizes were evaluated: 10, 25, and 100 neurons. The results indicated that the accuracies of the models with these three-layer sizes are quite similar, as shown in Figure 6. The network with a layer size of 25 neurons demonstrated marginally higher accuracy. However, since the three layer sizes offer almost the same level of accuracy, the layer size of 10 has been selected as the candidate due to its simplicity.

**Figure 7:**
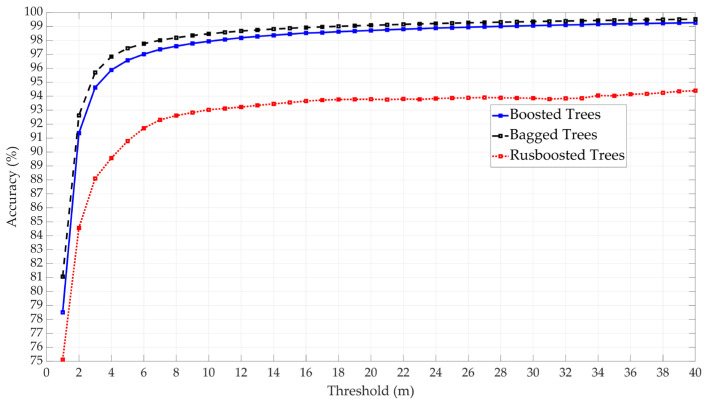
Comparative performance of three ensemble classifier models: Boosted Trees, Bagged Trees, and RUSBoost.

In the testing of ensemble classifiers, three options were evaluated: Boosted Trees, Bagged Trees, and Random Under Sampling (RUSBoost) Trees, utilizing 30 learning cycles and a learning rate of 0.1. The results indicated that Bagged Trees provided the best accuracy, followed by Boosted Trees, as shown in Figure 7. Thus, Bagged Trees have been selected as the candidate for the ensemble classifiers.

**Figure 8:**
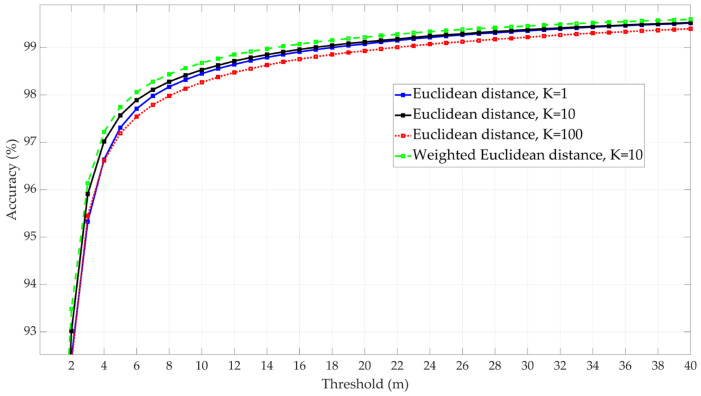
Performance comparison of four KNN models: non-weighted with 1, 10, and 100 neighbors (k) and weighted with 10 neighbors.

For the KNN models, tests were conducted using non-weighted Euclidean distance-based models with three numbers of neighbors: 1, 10, and 100. The results showed that the model achieved the best accuracy with 10 neighbors. In addition, a weighted KNN model using 10 neighbors was tested and assessed against the non-weighted one, as the results show an increase in model accuracy with the weighted approach. Figure 8 presents the results for the four KNN models. The weighted KNN model using 10 neighbors has been selected as the candidate KNN model.

**Figure 9:**
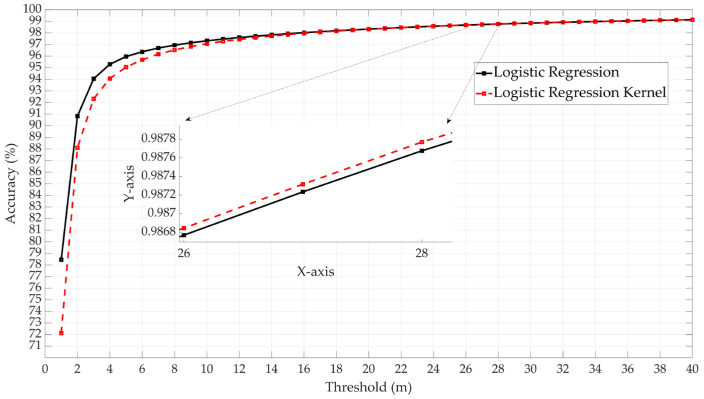
Comparison of Logistic Regression and Logistic Regression Kernel models.

**Figure 10:**
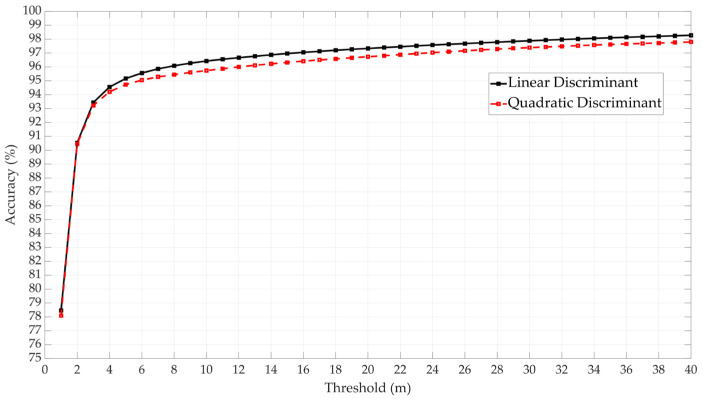
Performance evaluation of Linear and Quadratic Discriminant approaches.

In the logistic regression approach, both Logistic Regression and Logistic Regression Kernel have been tested. The former showed higher performance, as shown in Figure 9. In the Discriminant Analysis approach, both Linear and Quadratic Discriminant methods were tested. The Linear approach demonstrated higher performance, as shown in Figure 10.

In summary, this section selects the configuration parameters for the six ML approaches, including a decision tree model with a maximum of 4 splits, a neural network with a layer size of 10, Bagged Trees, a weighted KNN using 10 neighbors, Logistic Regression, and Linear Discriminant Analysis. The following section will comprehensively compare the selected models.

### 3.3. Model Comparison

**Figure 11:**
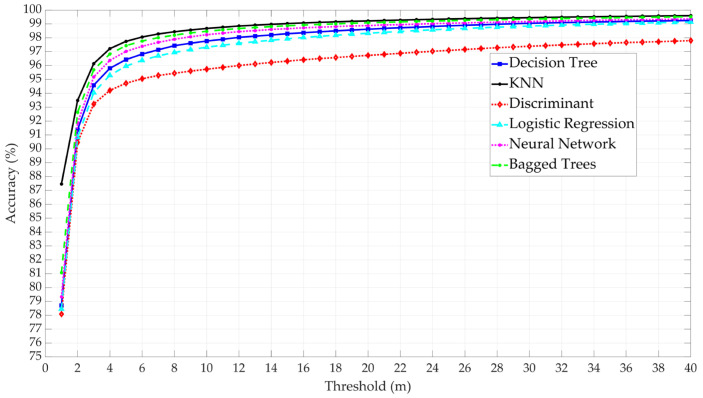
Comparative analysis of the six ML model performance based on model accuracy.

The results show that KNN provides the highest accuracy, followed by Bagged Trees, NN, Decision Tree, Logistic Regression, and Discriminant Analysis, as shown in Figure 11. The results indicate that the ML models can provide a high level of prediction accuracy, especially KNN, Bagged Trees, and NN, which offer higher than 95% accuracy when the threshold is equal to or greater than 4 m. In general, the accuracy dramatically increases from 1 to 2 m for all six ML models, but the rate of increase becomes lower after 2 m and gradually diminishes with higher thresholds. In the case of Weighted KNN, for example, the rate of increase becomes less than 0.005 per step (1 m) after the 4-m threshold and further decreases to less than 0.001 per step after the 9-m threshold.

**Figure 12:**
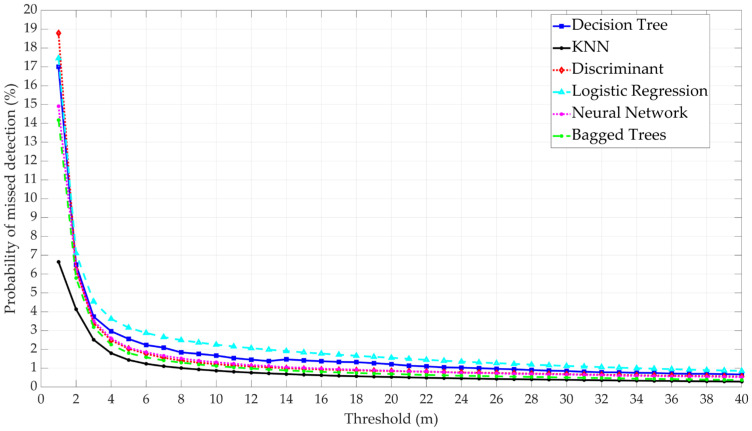
Comparative analysis of the six ML model performances based on the probability of missed detection.

The probability of missed detection results shows that the ML model with the lowest probability of missed detection is KNN, followed by Bagged Trees, NN, Decision Tree, and Logistic Regression, respectively, as shown in Figure 12. These models also have the highest validation accuracy. Put differently, the models with the highest accuracy are also the ones with the lowest probability of missed detection, which are recommended for implementation in safety-of-life applications. For KNN, the model with the lowest probability of missed detection, the results show that the probability of missed detection varied between $3\times 10^{-3}$ and $7\times 10^{-2}$ based on the selected threshold.

**Figure 13:**
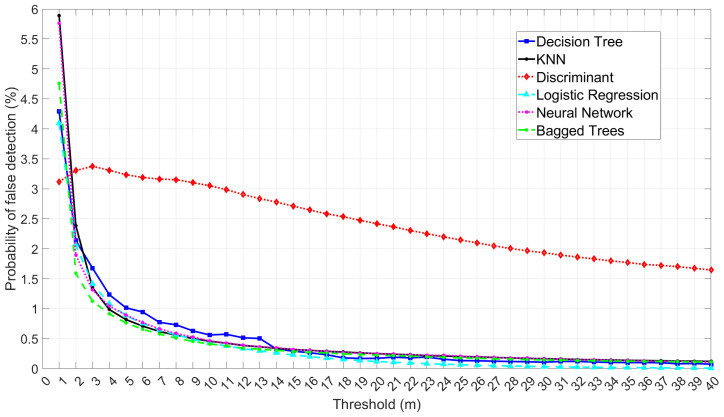
Comparative analysis of the six ML model performances based on the probability of false detection.

The probability of false detection results indicates that the best ML models offering the lowest false detection probability vary depending on the selected threshold, as shown in Figure 13. Generally, Bagged Trees and Logistic Regression have the lowest probability of false detection, followed by NN, KNN, and Decision Tree. Meanwhile, Discriminant Analysis exhibits the highest false detection probability at thresholds between 2 to 40 m, although it shows the lowest probability at a threshold of 1 m. Bagged Trees have the lowest probability of false detection in the 2 to 11 m range, while Logistic Regression shows the lowest probability from a threshold of 12 to 40 m. These results show that although KNN has higher accuracy and a lower probability of missed detection than Bagged Trees, it has higher false detection percentages, which leads to the loss of more clean data. This suggests using Bagged Trees rather than KNN when the target is to reduce the rate of losing clean data.

By comparing accuracy, probability of missed detection, and probability of false detection, the results reveal a key trade-off: reducing the threshold increases data quality but decreases prediction accuracy, leading to a higher percentage of lost high-quality data and a greater probability of missed detection. As the threshold increases, the detection process becomes less stringent, improving the model’s performance and accuracy. In addition, the QIs exhibit a stronger correlation with potential failures, suggesting that model accuracy improves as the threshold increases. This improvement results in a simultaneous decrease in the probability of false detection and the probability of missed detection, as these error probabilities are inherently linked.

**Figure 14:**
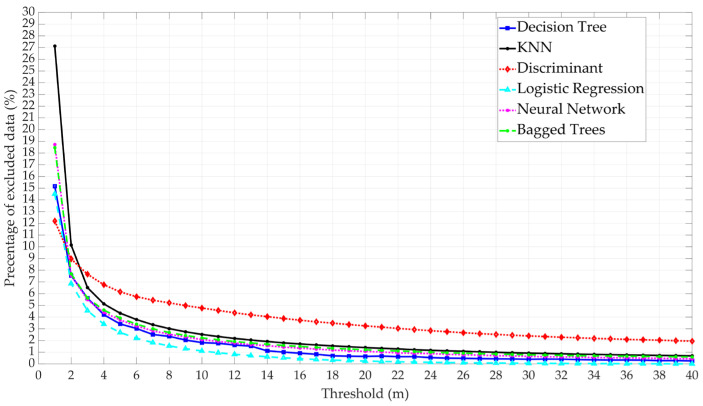
Comparative analysis of the six ML model performance based on the percentage of excluded data.

The percentage of excluded data shows that this percentage varies between 0.01% and 27% depending on the models and threshold selection, as shown in Figure 14. An exclusion rate of 27% is considered high, as losing more than a quarter of the satellites in the early stages of processing may impact model performance. This is especially significant considering the FDE process within the RAIM algorithm, which further excludes data. Moreover, excluding satellites can affect satellite geometry, particularly since satellites with high elevation angles have a higher probability of larger range errors. Therefore, selecting the threshold should strike a balance between the exclusion percentage and satellite geometry, then the positioning performance.

Comparing the results reveals that the 2–7-m threshold range is generally reliable. In this range, the ratio of excluded data varies from 2% to 9% of the datasets, likely leading to higher data quality than the 7–40-m threshold. In addition, within this range, the probability of missed detection is relatively low, offering a solid foundation for RAIM algorithms. However, the threshold should be precisely defined based on the requirements and by evaluating its impact on the positional domain.

In summary, the results demonstrate that ML models can provide a high level of prediction accuracy for outlier detection, exceeding 95% after a 2-m threshold and 99% after a 13-m threshold. The results also show that the model with the lowest probability of missed detection, which is a main concern for mission-critical applications, is also the model with the highest accuracy, and not always the model with the lowest false detection probability. This suggests that model selection should be based on four aspects: model accuracy, probability of missed detection, probability of false detection, and percentage of detected outliers. The results identify four candidate models that can be used for the FDE process: KNN, Bagged Trees, NN, and Discriminant Analysis. The selection among these models can vary based on application requirements.

## 4. Conclusions

The FDE process is of special importance for GNSS mission-critical applications. This paper has utilized six ML models for the FDE process at the user level and measurement domain, including Decision Tree, KNN, Discriminant, Logistic, Neural Network, and Trees (Boosted, Bagged, and RUSBoosted). A sensitivity analysis has been conducted for thresholds used to label faulty measurements across 40 thresholds (ranging from 1 m to 40 m). The six ML models have been assessed against these 40 threshold values in four aspects: accuracy, probability of missed detection, probability of false detection, and the percentage of detected outliers.

The results show that the ML models can provide a high-performance FDE process in the measurement domain, achieving more than 95% accuracy when the pseudorange failure threshold is equal to or greater than 3 m.

The results in this paper suggest utilizing ML for the FDE process in the measurement domain within the positioning algorithm to support various applications such as aviation, maritime, drones, autonomous driving, and farming. This can simplify the complexity of ARAIM algorithms and reduce the protection level regions, leading to improved system performance.

However, since the best model choice depends on data characteristics and may vary based on the environment, future work should investigate the optimal ML models to be utilized in various environments. In addition, future work should consider the computational complexity of these models in real-time applications. Furthermore, future research should develop an ML-based FDE at the system level and an ML-based FDE for the carrier phase at both the user and system levels.

## Figures and Tables

**Table 1:** QI mean and standard deviation values.

QI	Units	Mean	Standard Deviation
C/N0	dBHz	41	6.6
Code Lock Time	s	505	556
MP STDEV	m	1.50	10
Phase Lock Time	s	74	145
Elevation	Degrees	37.7	21
Speed	m/s	19.4	11.9

**Table 2:** Overview of categorical QIs (categories and their respective percentage distributions).

QI	Categories	Percentage	QI	Categories	Percentage
GNSS ID	GPS	52.7	Code Lock	Not Achieved	2.6
	GLONASS	47.3		Achieved	97.4
Signal ID	GPS L1	33.0	Phase Lock	Not Achieved	7.8
	GPS L2CL	18.8		Achieved	92.2
	GPS L2CM	1.0	Half Cycle Valid	Invalid	14.5
	GLONASS L1 OF	24.9		Valid	85.5
	GLONASS L2OF	22.4			

**Table 3:** Pearson correlation results.

	GNSS ID	Signal ID	C/N0	Code Lock	Code Lock Time	MP STDEV	Phase Lock	Phase Lock Time	Half Cycle Valid	Elevation	Speed
GNSS ID	1										
Signal ID	−0.08	1									
C/N0	−0.21	−0.39	1								
Code Lock	0.10	−0.07	0.10	1							
Code Lock Time	0.19	0.03	0.07	0.14	1						
MP STDEV	0.07	−0.02	−0.11	−0.02	−0.02	1					
Phase Lock	−0.03	−0.04	0.54	0.13	0.03	−0.06	1				
Phase Lock Time	−0.03	0.01	0.16	0.08	0.24	−0.04	0.15	1			
Half Cycle Valid	−0.05	−0.05	0.52	0.28	0.08	−0.08	0.71	0.21	1		
Elevation	0.00	0.03	0.42	0.10	0.17	−0.07	0.19	0.15	0.25	1	
Speed	0.01	0.01	0.04	−0.03	−0.08	−0.03	−0.03	−0.47	−0.05	0.01	1
Pseudorange Error	0.01	−0.01	−0.08	−0.02	0.00	0.02	−0.07	−0.01	−0.05	−0.03	−0.02

## Data Availability

The data utilized in this study were provided courtesy of u-blox.
